# Development and Use of Assistive Technologies in Spinal Cord Injury: A Narrative Review of Reviews on the Evolution, Opportunities, and Bottlenecks of Their Integration in the Health Domain

**DOI:** 10.3390/healthcare11111646

**Published:** 2023-06-04

**Authors:** Giovanni Morone, Antonia Pirrera, Antonio Iannone, Daniele Giansanti

**Affiliations:** 1 Department of Life, Health and Environmental Sciences, University of L’Aquila, 67100 L’Aquila, Italy; 2 San Raffaele Institute of Sulmona, 67039 Sulmona, Italy; 3 Centre TISP, Italian Institute of Health, 00161 Roma, Italy; 4 Association “Relazionarti”, 05100 Terni, Italy; 5 CREA Italian National Research Body, 00184 Roma, Italy

**Keywords:** assistive technology, medical device, spinal cord injury, disability

## Abstract

Assistive technologies are increasingly taking a leading role in supporting people with spinal cord injury (SCI). This narrative review of reviews intends to contribute by making a map point investigating the integration of ATs in SCI. The methodology of the review was based on: (I) a search of PubMed and Scopus and (II) an eligibility assessment using specific parameters. The outcome highlighted the following: -The evolution of ATs considered in the context of SCI, considering ATs as both products and/or services in standalone and/or networked devices, and as processes of delivery. -Innovative technologies could play an important role in improving the quality of life and in minimizing costs in healthcare. -The international scientific community has identified ATs as one of the six strategic development areas in SCI. The overview also allowed the detection of some problems: (I) The ethical and regulatory aspects have been addressed in a weak way and only in specific and limited cases. (II) There is a lack of studies on the use and applications of ATs in SCI with a focus in multiple domains (e.g., costs, acceptance, dissemination, problems, regulatory aspects, ethical aspects, and other issues important for integration into the health domain). This review highlights the need for further studies and activities focused on integrating consensus in multiple domains, including ethics and regulations, to aid researchers and decision-makers in the field.

## 1. Introduction

This study’s scope is located within the area of neuromotor and sensory disabilities, with a particular focus on those associated with spinal cord injury (SCI). These disabilities have a significant impact on the independence and quality of life of those affected. The spinal cord is a bundle of nerves that sends information between the brain and the rest of the body. A spinal cord injury can happen due to a traumatic event and can cause a reduction or absence of movement, sensation, and function of body organs below the level of the injury [1,2,3,4]. The severity and location of the injury can result in a range of symptoms from pain or numbness to paralysis and incontinence. The outcome varies from complete recovery to irreversible disability, and complications can occur in both the short and long term after the injury. Complete SCI means the spinal cord is irreversibly compromised, whereas incomplete SCI means the person retains some motor and/or sensory function below the site of injury. On average, in the US [2], there are around 12,000 (40 cases per million inhabitants) SCIs. The main causes are road accidents (48%), falls (16%), sports accidents (10%), trauma from aggression, such as with a firearm (12%), and accidents in the workplace. It should be considered that the higher the damage is positioned on the spine, the more serious the paralysis is likely to be. Table 1 provides an overview of the correlation between the location of the lesion and the resulting impairment in function in the case of tetraplegia. Assistive technologies (ATs) enable individuals with disabilities to live healthy, productive, independent, and dignified lives, and to participate in education, the labor market, and civic life [5]. Without these technologies, people with disabilities may face exclusion and isolation, leading to poverty and an increased impact of disease and disability on individuals, families, and society. Over time, technological aids for the disabled have improved in regard to accessibility, evolution, and perception, both in terms of common opinion and institutional viewpoints [5,6]. As society continues to age and grow, assistive technologies play an increasingly important role in improving the quality of life, access to resources and life activities, and work opportunities for individuals with disabilities. Assistive technologies must be tailored to individual problems, ranging from muscle weakness to the inability to breathe, move the head, and speak [7,8,9]. ATs provide a great opportunity to support people with different typologies of disabilities. It is therefore important to address the issue of their development, integration, and use in the SCI population.

The integration of assistive technologies (ATs) with SCI has been a topic of research since 1994 [10], with advancements in technology such as robotics, virtual reality, Artificial Intelligence (AI), brain–computer interfaces (BCIs), and miniaturized electronics offering opportunities to improve support for individuals with disabilities. However, to fully realize these benefits, several issues need to be addressed, including developing algorithms and introducing regulations for stable applications in the health domain. 

Reviews play an important role in categorizing and cataloguing themes emerging from different experiences in scientific fields, serving as a practical tool for both scientists and decision-makers, and paving the way for stabilizing the scientific field. Reviews also provide preliminary scientific evidence for the design of agreement initiatives, guidelines, Health Technology Assessment reports, Comparative Assessment Reports, or Consensus Conferences.

The purpose of this overview is to analyze how the published reviews are moving around the field of the design and integration of ATs with SCI treatment and support, and therefore to answer the following question: “*How is the integration of ATs and the SCI population described in the reviews*”?

Secondary aims of this overview include:

- Analyzing the movement and trends of published reviews in the field of the design and integration of ATs with SCI.
- Answering the question of how the relationship between ATs and SCI is addressed in these reviews.
- Identifying the themes that scientists are dedicating the most attention to.
- Recognizing the current research trends in this newly developed scientific sector.
- Providing insight into research directions and identifying gaps and bottlenecks in the field.

## 2. Methods

To ensure consistency, we employed a Standard Narrative Checklist, specifically designed for reviews falling under the narrative category (available online at: [11]). Since our investigation focused on assistive technologies in this field, we conducted targeted searches on Pubmed and Scopus for relevant reviews. Our overview comprised components that exclusively explored the aforementioned topic.

The composite key used in the search is reported in Box 1.

Box 1 **The proposed composite key.**

*(“assistive technology”[Title/Abstract] OR “assistive technologies”[Title/Abstract]) AND (“spinal cord injury”[Title/Abstract] OR “paraplegia”[Title/Abstract] OR “tetraplegia”[Title/Abstract] OR “quadriplegia”[Title/Abstract] OR ((“paralysing”[All Fields] OR “paralysis”[MeSH Terms] OR “paralysis”[All Fields] OR “paralyse”[All Fields] OR “paralysed”[All Fields] OR “paralyses”[All Fields]) AND “of lower limbs”[Title/Abstract]) OR ((“paralysing”[All Fields] OR “paralysis”[MeSH Terms] OR “paralysis”[All Fields] OR “paralyse”[All Fields] OR “paralysed”[All Fields] OR “paralyses”[All Fields]) AND “of upper limbs”[Title/Abstract]))*

This overview was carefully crafted with a consideration of five parameters (N1-N5) that have been evaluated on a scale ranging from one (minimum) to five (maximum). In addition to these parameters, there is also a binary assessment (N6: yes/no). The parameters are as follows:

*N1: Clarity of introduction and rationale for the review*.

*N2: Appropriateness of review design*.

*N3: Clear description of methods*.

*N4: Clear presentation of results*.

*N5: Justification of conclusions based on results*.

*N6: Full disclosure of potential conflicts of interest by authors*.

These parameters have been thoughtfully selected to ensure the comprehensiveness and quality of this overview.

## 3. Results

At the time of this overview, the search using the composite key yielded 220 relevant papers in total, out of which 27 were reviews (listed as [12,13,14,15,16,17,18,19,20,21,22,23,24,25,26,27,28,29,30,31,32,33,34,35,36,37,38]); the remaining 193 were scientific papers. The field of study in question emerged roughly 30 years ago, circa 1993, with the first review article published in 1999. All selected reviews met the criteria of having a “Yes” result for parameter N6, and a score of at least three for parameters N1 to N5. 

The results of the overview were organized in such a way as to report both the expectations of the first studies and the emerging themes in the more recent reviews. This was done in order to retrace expectations on the one hand, and on the other, to highlight developments in this area.

The first paragraph, Section 3.1 *“Assistive Technologies for Spinal Cord Injuries: A Review of Pioneering Research*”, therefore provides a review of the pioneering research.

The second paragraph, Section 3.2 *“Emerging and Consolidated Themes in the Reviews: A Comprehensive Analysis*”, is divided into four sub-paragraphs (Section 3.2.1, Section 3.2.2, Section 3.2.3 and Section 3.2.4) relating to emerging themes.

Sub-paragraph Section 3.2.1 “*Unlocking the Potential of Assistive Technologies: A Comprehensive Analysis of Intervention Areas and Determinants*” reports studies that have focused on the areas of intervention and on the health determinants related to Ats.

Sub-paragraph Section 3.2.2, “*Economic Impacts, Social Acceptance, and Quality of Life: An Analysis*” reports studies dedicated to the important aspects related to the integration and acceptability of Ats in the health domain.

Sub-paragraph Section 3.2.3, “*Ethics and Regulatory Compliance*” is dedicated to the ethical and regulatory aspects, as dealt with by the studies.

Sub-paragraph Section 3.2.4, “*Advancing the Integration of Assistive Technologies in Spinal Cord Injury through Technical Innovation*” reports the studies dedicated to the contribution of technological innovation in the field of AT.

### 3.1. Assistive Technologies for Spinal Cord Injuries: A Review of Pioneering Research

An initial analysis of early reviews sheds light on the expectations of scholars regarding the development of assistive technologies (ATs) and whether those expectations have been met. Overall, the first available reviews were optimistic about the potential of ATs in this field, citing several key factors: devices for passive standing [33], advancements in sensors, control electronics, equipment, and telecommunications due to bioengineering and telehealth [29,31], and the expected growth of neural interface systems (NISs) for individuals with paralysis [30]. Other authors also predicted that this technological progress would lead to greater accessibility and affordability of ATs [32].

The review by Gear et al. in 1999 [33] concentrated on the technological innovations in rehabilitation medicine, with a focus on the potential of devices for passive standing to address chronic immobilization pathologies in individuals with spinal cord injury. According to the authors, these devices could offer significant benefits, such as reducing decubitus plagues, preventing demineralization, minimizing cardiovascular issues, and maintaining muscle tone through the upright position they facilitate.

Cooper et al. in 2004 [31] identified significant opportunities for scientific and economic growth in the field of bioengineering for SCI support, with advancements in control, sensor, instrumental electronics, and telecommunications expected to play a significant role. 

Donoghue et al. in 2007 [30] envisioned NISs as promising technologies with potential applications in support interfaces, neurological and sensory rehabilitation, diagnosis, management, and in the construction of medical knowledge around brain function. 

Bendixen et al. [29] evaluated the usefulness of telehealth as an encouraging method for daily monitoring and rehabilitation in SCI in a 2009 study, citing successful experiences in applying telehealth models to complex polytrauma cases in combat-wounded individuals. 

Finally, McKinley et al.’s 2004 study [32] addressed the cost of ATs, highlighting the affordability of many available options that can provide essential support to individuals with SCI.

Table 2 summarizes the highlights in the pioneering studies.

### 3.2. Emerging and Consolidated Themes in the Reviews: A Comprehensive Analysis

The reviews in the range of [12,13,14,15,16,17,18,19,20,21,22,23,24,25,26,27,28,29,30,31,32,33,34,35,36,37,38] explored a variety of issues related to ATs, such as the intervention areas [13], the determinants of health and the key factors [34], and innovative technological integration. Many of these reviews focused on the effectiveness of ATs, including robotics, Functional Electric Stimulation (FES), and brain–computer interfaces (BCIs) in clinical rehabilitation [12,15,17,18,20,23,24,25,26,27,28,35,36,38]. The economic impact, acceptance, and quality of ATs were also examined [16,19,22,36,37], along with ethical and regulatory considerations [12,21]. In addition to products such as robotics and FES, some reviews also studied the assignment processes of ATs. For example, managing databases of stem cells for SCI were considered to be ATs [14]. This aligns with the World Health Organization’s definition of AT, which includes services that aid in the delivery of assistive products beyond the products themselves [39]. Overall, this research suggests that ATs have enormous potential to support clinical rehabilitation, through both innovative products and services.

#### 3.2.1. Unlocking the Potential of Assistive Technologies: A Comprehensive Analysis of Intervention Areas and Determinants

The clear assignment of the intervention area in international consensus initiatives and the identification of health determinants influencing the acceptance of an AT are key factors. Two studies [13,34] (Table 3) addressed these issues separately. 

The study conducted by Morse et al. [13] presented the consensus findings of the participants at the SCI 2020 conference, which included regulators, researchers, clinicians, healthcare professionals, stakeholders, patients, caregivers, and persons with SCI. The study highlighted six areas that were identified (literary quotation): “(1) opportunities in the acute post-injury phase; (2) innovating repair, plasticity, and regeneration in the subacute and chronic periods; (3) with us, not for us: community activity and priorities; (4) neuromodulation to improve neurological function months and years after SCI; (5) health and secondary health effects of chronic SCI; and (6) technological facilitation, prosthetic and robotic interventions and therapies across the spectrum of mild/moderate/severe”. Interestingly, ATs were found to have a specific dedicated area, i.e., domain 6, while also playing a crucial role in domain 1 and domain 2.

Gurung et al. [34] investigated the determinants of health correlated to ATs with particular attention paid to the modifiable factors that impacted the health of community-dwelling people with SCI. The review found that socio-structural factors (such as social attitudes, health care access, information access, and funding and policies) and environmental factors (including built environment, housing, transportation, ATs, and natural environment) impacted people with SCI. Future research should investigate the effects of these modifiable factors using qualitative methods and community-based participatory research, and considering individual characteristics and resources.

#### 3.2.2. Economic Impacts, Social Acceptance, and Quality of Life: An Analysis

Five studies addressed the economic impact, acceptance, and quality of life, or service aspects related to ATs, for people with SCI [16,19,22,36,37] (Table 4). One study by Gallagher et al. [16] focused on the wheelchair and its relationship with people with SCI, examining aspects such as satisfaction, performance, and participation. They found that the implementation and seating of the AT affected the ability of people with SCI to participate equally in society and highlighted the need for international policies to assure equal access to resources and investigations covering multiple domains.

Another study by Baldassin et al. [19] specifically investigated how personal-computer-based ATs could improve the quality of life of people with SCI. Many studies suggested that these ATs could improve independence, participation, and self-esteem. Orejuela-Zapata et al. [37] collected and discussed the main needs, expectations, and barriers of people with quadriplegia and caregivers in relation to the self-help devices that are currently used for daily tasks. The major advantages, disadvantages, and challenges of the existing ATs were exposed and discussed in order to evaluate whether an existing technology could be combined with others to expand its scope, enhance its performance, or solve its limitations, with the aim of improving the adherence of the quadriplegic population to these technologies and enhancing their quality of life.

Readioff et al. [36] reported that there was no clear clinical consensus on the effectiveness of the current ATs for the cervical SCI population at the time of their study. They highlighted that ATs could improve functions of the upper limbs in SCI patients, but that it was challenging to draw generalizable conclusions because of a lack of investigations covering multiple domains. 

Finally, Miller et al. [22] focused on costs and the potential positive impact of ATs on physical activity levels for people with SCI in the US. They found that investing in specific rehabilitation protocols and ATs could improve health conditions and minimize costs for people with SCI.

Overall, these studies showed the importance of ATs in improving the lives of people with SCIs and highlighted the need for continued research, investment in this area, and targeted insights into multiple domains of intervention.

#### 3.2.3. Ethics and Regulatory Compliance

Only two literature reviews tackled ethical and regulatory concerns, but they focused on specific sectors rather than addressing them on a general level [12,21] (Table 5). Burwell et al. [21] discussed the ethical implications of using brain–computer interfaces (BCIs) as ATs in SCI patients, highlighting the challenges that arise from the direct connection to the brain. They identified several ethical, social, and legal issues concerning personhood, stigma, autonomy, privacy, research ethics, safety, responsibility, and justice. However, they concluded that few international recommendations have been developed to address these challenges. Meanwhile, Pirrera et al. [12] explored the regulatory complexities of tongue-barbell-piercing-based ATs. These complexities are very common in highly technologically innovative systems in the health sector, such as robotics and diagnosis using artificial intelligence. The authors [12] highlighted the potential regulatory frameworks applicable to these systems for alternative and augmentative communication based on the tongue piercing, emphasizing the intricate regulatory processes involved.

#### 3.2.4. Advancing the Integration of Assistive Technologies in Spinal Cord Injury through Technical Innovation

The integration of assistive technologies (ATs) was subjected to numerous investigations, with 14 studies delving into their technological effectiveness, specific applications, and clinical targets [12,15,17,18,20,23,24,25,26,27,28,35,36,38].

Table 6 reports the detected contributions of the technologies in ATs.

Readioff et al. [36] reviewed the literature on ATs developed to help individuals with SCI at the cervical level and categorized the ATs into five types of ATs: neuroprostheses, orthotic devices, hybrid systems, robots, and arm supports. Pirrera et al. [12] highlighted the state of the art of ATs based on the tongue barbell piercing, emphasizing their high acceptance in mechatronics integration, particularly in quadriplegia patients with severe movement limitations. Klein and Baumeister [15] analyzed mechatronics and robotics as ATs for food administration, highlighting their limited use also in the most developed countries. Clark et al. [17] advocated for the integration of virtual reality (VR) and robotics for rehabilitation to increase its efficacy. Fridén and Lieber [18] suggested the use and integration of ATs, such as FES, for upper extremity recovery after surgeries in tetraplegia. Vibhuti et al. [35] also focused on VR and investigated the effectiveness of home-based exercise treatment for individuals with neuromotor impairments. The study concluded that home-based systems could provide efficacious therapy and facilitated the development and integration of better methods for rehabilitation. Palermo et al. [20] focused on powered exoskeletons (PEXOs) for clinical applications, highlighting their potential to minimize secondary medical complications in SCI patients. Lajeunesse et al. [23], on the other hand, showed skepticism regarding the performance of PEXOs for mobility among SCI patients. Tung et al. [24] focused on ATs for pressure ulcer prevention in SCI, identifying specific categories supporting self-management but pointing out low-to-moderate effectiveness. Rup highlighted BCI’s promise, though further specific studies are necessary to gauge its applicability in the clinical setting [25]. Charters et al. [26] reviewed electronic portable assistive devices in SCI and suggested the use of portable electronic reminders as a practice guideline. Bryden et al. [27] focused on youth and identified key ATs, including therapeutic and functional stimulation, EMG biofeedback, and ATs for access to the computer. Kalsi-Ryan and Verrier [28] presented a study that found FES therapies useful and valuable during the subacute phase of recovery for persons with disability caused by quadriplegia. Lastly, Athanasiou et al. [38] discussed the impact of spinal cord injury on brain connectivity and organization. Whereas changes in brain structure have been extensively studied, knowledge regarding brain connectivity following SCI is lacking. This, according to the authors, could affect to the proper choice of AT.

Overall, the studies (Table 6) suggest that these technologies can help individuals with disabilities to manage their daily lives, prevent complications, and enhance their mobility and rehabilitation. The overview highlights that there is a wide variety of assistive technologies (ATs) available for different clinical targets and specific applications, with potential applications in pressure ulcer prevention, rehabilitation, food administration, youth therapy, spinal cord injury, and brain connectivity. These technologies include neuro-prostheses, orthotic devices, hybrid systems, robots, arm supports, tongue barbell piercings, mechatronics, robotics, virtual reality, functional electrical stimulation, powered exoskeletons, brain–computer interfaces, and electronic portable assistive devices. The effectiveness of some of these technologies varies, and further specific studies are necessary to gauge their applicability in the clinical setting. However, many of these technologies have shown potential to improve the lives and wellbeing of individuals with disabilities. Further research is required to determine their optimal effectiveness and applicability in clinical settings.

## 4. Discussion

### 4.1. Highlights

Studies on ATs used in SCI have a rich history of about thirty years [10]. Reviews play a crucial role in creating Evidence-Based Medicine, which is necessary for integrating and consolidating medical practices in the health domain. This narrative review of reviews aimed to contribute by mapping the key themes related to the introduction of ATs in this field, reporting opportunities and problems.

The integration of ATs used in SCI within the health domain is a complex and challenging task. Based on the evolution of the term [39], ATs can include various technologies, such as products with high mechatronics, information technology, services, and processes for the telematic distribution of information. ATs can work alone or as part of a digital network. Therefore, when addressing the integration of ATs used in SCI in the health domain, three crucial issues must be considered. 

*First*, if an AT works in a digital network, it inherits the complex problems of (i) telemedicine, (ii) electronic health, and (iii) mobile health, and includes regulation, ethics, and the need for stability in routine applications [40,41]. It is evident that, depending on the applications in a digital network, an AT can belong (reporting non-exhaustive examples) to: -(i), the case of telerehabilitation [29]; -(ii), the case of stem cell database services for ATs in multiple sclerosis [14]; -(iii) the case of systems based on the tongue barbell piercing [12].

*Second*, when an AT works alone, the peculiarities of the technologies and the methods of application must be considered. 

*Finally*, when an AT must be provided, implications at the national system level come into play. A thorough analysis of emerging issues with reference to these three points is essential when addressing the integration process in the health domain.

The expectations from the first studies (Table 2) on the development of ATs included the potential for devices for passive standing [33] to address chronic immobilization pathologies in individuals with spinal cord injury (SCI). Advancements in sensors, control electronics, equipment, and telecommunications due to bioengineering were also expected to play a significant role [31]. NISs for individuals with paralysis were also considered promising technologies with potential applications in support interfaces, neurological and sensory rehabilitation, diagnosis, management, and the construction of medical knowledge around brain function [30]. Additionally, there was a prediction that this technological progress would lead to the greater accessibility and affordability of ATs through telehealth as an encouraging method for daily monitoring and rehabilitation in SCI [32]. Overall, the initial reviews were optimistic about the potential of ATs in this field. Almost all expectations have been met. 

However, compared to the forecasts, the costs of ATs have not decreased as expected, and this causes diffusion problems. For example, in the poorest countries [42], NISs have presented critical issues in the realm of BCIs, as highlighted in more recent studies [12,21,25].

The overview highlighted the importance of considering both intervention areas and health determinants (Table 3) when designing and implementing ATs for people with SCI [13,34]. It is important to engage a diverse range of stakeholders, including people with SCI, in the development and implementation of ATs to ensure they meet the needs of the community [13]. Additionally, the impact of socio-structural and environmental factors should be considered in conjunction with individual characteristics and resources [34]. These findings can inform the development of more effective ATs and improve the overall health and wellbeing of people with SCI.

The opportunities for the technological development (Table 6) of the use of assistive technologies (ATs) are vast and varied, as demonstrated by the numerous studies that investigated their effectiveness, applications, and clinical targets. The technologies were shown to play an important role. The technologies highlighted in the studies include targets [12,15,17,18,20,23,24,25,26,27,28,35,36,38], neuro-prostheses, orthotic devices, hybrid systems, robots, arm supports, tongue barbell piercings, mechatronics, robotics, virtual reality, functional electrical stimulation (FES), powered exoskeletons (PEXOs), brain–computer interfaces (BCIs), and electronic portable assistive devices. These technologies have potential applications in assistance, pressure ulcer prevention, rehabilitation, food administration, youth therapy, and brain connectivity. The studies highlighted the efficacy, acceptance, and value of using ATs as therapeutic interventions and for self-management in individuals with SCI. The reviews demonstrated that:

- The advancement and increasing use of ATs present an opportunity for improving the quality of life of individuals with disabilities.
- Different types of ATs have potential applications in various clinical targets, such as assistance, pressure ulcer prevention, rehabilitation, food administration, youth therapy, and brain connectivity.
- Home-based systems, virtual reality, and electronic portable devices present opportunities for effective therapy and better management of neuromotor disorders.
- FES, PEXOs, and BCIs have been found to be useful and valuable as ATs and therapeutic interventions during the recovery phase for persons with disability caused by quadriplegia.

However, some critical issues and needs for further study have also emerged in specific sectors. For example:

- Skepticism exists regarding the performance of PEXOs for mobility among SCI patients [23].
- A low-to-moderate effectiveness of ATs for pressure ulcer prevention in SCI has been identified [24].
- There are limits in the application of BCIs [25].
- There is a lack of knowledge regarding brain connectivity following SCI, which could impact the proper choice of ATs [38].
- Mechatronics and robotics as ATs for food administration have limited also in the most developed countries [15].

The overview investigated the economic impact, acceptance, quality of life, service aspects, and effectiveness of assistive technologies (ATs) for people with SCI [16,19,22,36,37]. The studies focused on various ATs such as wheelchairs, personal-computer-based ATs, and self-help devices. The studies suggested (Table 4) that ATs could improve independence, participation, self-esteem, functions of the upper limbs, physical activity levels, and overall quality of life of people with SCIs. However, the effectiveness and generalizability of ATs remain uncertain, and more research, investment, and targeted insights into multiple domains of intervention are necessary to ensure equal access to resources and improve the lives of people with SCIs.

The existing literature on ethical and regulatory concerns in assistive technology is limited and was focused on specific sectors or technologies [12,21] (Table 5). There is a need for a comprehensive exploration of the ethical and regulatory challenges associated with ATs on a general level. Such research could help policymakers to develop appropriate regulatory frameworks and guidelines to ensure the safe and ethical use of ATs for the benefit of all users.

### 4.2. Emerging Criticisms and Need for Further Research

The overview showed, in addition to some specific problems [15,23,24,25,38] categorized by type of AT (Table 6), some areas needed for further study in relation to both the ethical and regulatory aspects (Table 5) and technology assessments capable of covering multiple domains (Table 4). 

#### 4.2.1. Comparison with Recent Publications Focused on Ethics and Regulations

As highlighted in the results, ethical and regulatory aspects were addressed in some specific cases and in a limited way [12,21]. 

We conducted on Pubmed a search regarding the past three years that included all types of articles dealing with ethics and regulations to compare the trends found in the reviews. Despite the pivotal role played by ATs during this period, due to the pandemic, no studies focusing specifically on ethical and regulatory aspects were found. After conducting a search using the composite key in Box 2, position 1 and applying various filters, 10 relevant studies emerged. However, three of these were reviews that had already been covered in our overview. Out of the remaining seven, only five [43,44,45,46,47] touched upon the specific topic of interest (regarding ethics or regulation), and even then, this was only in a secondary or complementary manner, serving to confirm the trends seen in the overview of reviews. 

Armstrong-Wood et al. [43] found that individuals with SCI encountered difficulties accessing their smartphones due to motor impairment, which can limit their hand or finger movement. To overcome these barriers, they might rely on ATs, although these methods could result in compromising their privacy and independence. Kubiak and Sklar [44] reported that the internet presented an opportunity to reduce barriers to social participation and increase social integration for individuals with tetraplegia; however, race, ethnicity, and income inequities limited access to internet providers, computers, and ATs, especially for Black and Hispanic individuals. The study conducted by Kim et al. [45], based on a focus group, emphasized the significance of protecting the privacy and rights of vulnerable individuals, such as those with tetraplegia, from exploitation or harm. Lau and Moumbar [46] presented a preliminary protocol for the use of a Lower-Limb Robotic Exoskeleton, which could only be tested on healthy individuals due to regulations, raising ethical concerns regarding the exclusion of individuals with disabilities from involvement in the testing process. Yao et al. [47] testified that Filipinos with SCI faced significant challenges in accessing ATs, and that occupational justice was not fully achieved in this population. Further exploration of the experiences of individuals with SCI in using ATs, according to the authors, could help occupational therapists to identify ways to address these challenges and advocate for greater recognition of occupational rights.

#### 4.2.2. Comparison with Recent Publications Focused on Technology Assessment

The results also highlighted the lack of comprehensive studies that address the use and applications of ATs in multiple domains [16,36], and that include comparisons, costs, acceptance, dissemination, regulatory and ethical aspects, and other relevant issues for integration into the healthcare domain. 

We conducted on Pubmed a search regarding the past three years that included all types of articles dealing with these aspects to compare the trends found in the reviews. Despite the pivotal role played by ATs during this period, due to the pandemic, no studies focusing specifically on multiple domains were found. After conducting a search using the composite key reported in Box 2, position 2 and applying various filters, 14 relevant studies emerged. However, four of these were reviews that had already been covered in our overview and two were not pertinent. The other studies focused only on single domains [48,49,50,51,52,53,54,55], confirming the trends of the overview of reviews.

Box 2 **The used composite keys.**

*Search: ((assistive technology[Title/Abstract]) OR
(assistive technologies[Title/Abstract])) AND ((spinal cord
injury[Title/Abstract]) OR (paraplegia[Title/Abstract]) OR
(tetraplegia[Title/Abstract]) OR (quadriplegia[Title/Abstract]) OR (paralysis
of lower limbs[Title/Abstract]) OR (paralysis of upper
limbs[Title/Abstract])) AND ((ethics) OR (regulation) OR (rule) OR
(standard)) Filters: from 2020/4/7–2023/4/7 Sort by: Publication Date*

*Search: ((assistive technology[Title/Abstract]) OR
(assistive technolo-gies[Title/Abstract])) AND ((spinal cord
injury[Title/Abstract]) OR (paraple-gia[Title/Abstract]) OR (tetraplegia[Title/Abstract])
OR (quadriplegia[Title/Abstract]) OR (paralysis of lower
limbs[Title/Abstract]) OR (paralysis of upper limbs[Title/Abstract])) AND
((acceptance[Title/Abstract]) OR (consensus[Title/Abstract]) OR
(assessment[Title/Abstract]) OR (satisfaction[Title/Abstract])) Filters: from
2020/4/7–2023/4/7 Sort by: Publication Date*

Yurkewich et al. [48] investigated the feasibility and effectiveness of using an exoskeleton-supported rehabilitation program for people with hand impairments after stroke or SCI. The intervention was assessed using Goal Attainment Scaling (GAS), the Box and Block Test (BBT), the System Usability Scale (SUS), the Motor Activity Log, and the Fugl-Meyer Assessment. Results showed that the intervention was feasible and effective. The study proposed by Forslund et al. [49] examined the long-term effects of SmartDrive, a rear drive power assist device, on mobility, everyday activity, and shoulder pain among spinal-cord-injured manual wheelchair users. The results indicated a tendency toward decreased pain and increased satisfaction with performance and independence when using the device. The work presented by Ottoboni et al. [50]: -introduced MAIA, a multifunctional, adaptive, and interactive AI system for controlling assistive devices. -Explored the acceptability of MAIA to end users through semi-structured interviews with patients and caregivers. Factors such as motivation, ease of use, and personal factors like gender, fragility levels, and psychological aspects of body image also impacted the acceptability. Marquez et al. [51] analyzed the relationship between environmental factors present in parasports, such as attitudes, support, services, AT, and policies. The study also validated the Assistive Technology Device Predisposition Assessment (ATD PA-Br). Overall, the study suggested that technology and services should act as facilitators of parasport performance. Butzer at al. [52] investigated the acceptance of a robotic hand exoskeleton. User tests demonstrated that its low weight, unintrusive size, high wearing comfort, and appealing appearance contributed to user acceptance and usability in daily life. Thorsen et al. [53] assessed a non-invasive method, called myoelectrically controlled functional electrical stimulation (MeCFES), for improving the tenodesis grip of people with tetraplegia. Results from the IPPA (Individual Prioritised Problems Assessment) questionnaire indicated the issues people with tetraplegia hope to solve with a neuroprosthesis for the hand. The satisfaction resulted very high. Monforte et al. [54] explored the process of becoming a long-term wheelchair user by means of a case study. The case study was based on an innovative approach conjugating a posthumanist perspective and qualitative methods, such as interviews and observation. Rice et al. [55] investigated the effects of using an anterior tilt-in-space power seat function on the functional activities, physical health, and user satisfaction of power wheelchair users. Results showed significant improvements in the safety of meal preparation and functional reach in the vertical direction. However, participants found the safety equipment restrictive.

### 4.3. Emerging Recommendations

In-depth research on specific scientific articles also highlighted the trends that emerged in the overview of reviews regarding both the poor investigation of ethical and regulatory aspects (Table 5) and the absence of studies on technology assessments capable of covering multiple domains (Table 4). Concerted actions involving experts, international scientific societies, and stakeholders could be useful for tackling these strategic issues more decisively. Initiatives such as the Consensus Conference on robotic rehabilitation in Italy can provide shared documents on applications, organization models, training, regulations, and ethics [56,57]. This review highlights the need for further studies and activities focused on integrating consensus in multiple domains, including ethics and regulations, to aid researchers and decision-makers in the field.

### 4.4. Limitations of the Overview

The narrative overview has limitations. The review considered reviews in English and one in German (available in Pubmed). The reviews in different languages, not available in Pubmed or Scopus, were not considered. PubMed and Scopus databases were consulted, and only peer-reviewed papers were considered in the review.

Future work should focus on the detected themes, directly analyzing the articles published after the overview on the identified themes and updating the medical knowledge by theme. 

## 5. Conclusions

In conclusion, the overview highlighted, through an analysis of the reviews, the evolution of ATs in the context of SCI, identifying the emerging fields of interest, with consideration of opportunities and problems. Technologies have been highlighted as playing an important role in improving the quality of life and minimizing costs in healthcare. The international scientific community identifies Ats as one of the six strategic development domains in SCI. However, the ethical and regulatory aspects have been addressed in a weak way and only in specific and limited cases. Even the acceptance of AT devices has not been extended to all technologies. What is missing are studies on the use and applications of ATs in SCI with a focus in multiple domains. They must include comparisons, costs, acceptance, dissemination, problems, regulatory aspects, ethical aspects, and other issues important for their integration into the health domain. This review highlights the need for further studies and activities focused on integrating consensus in multiple domains, including ethics and regulations, to aid researchers and decision-makers in the field.

## Figures and Tables

**Table 1:** Mapping the relationship between lesion location and functionality in tetraplegia.

Area	Functionality of the Respiratory System	Functionality of the Neuromuscular System
C1–C4	Mechanical breathing is required	Arms are totally paralyzed
C5	Problems with coughing. Requests for support in removing the secretions are probable	Paralysis of the muscles of triceps, hands, and wrists is present
C6	The same as above	Paralysis of the wrist flexors, triceps, and hands
C7–C8	The same as above	There is a difficulty in releasing and grasping and some force lacking in the muscles of the hands

**Table 2:** Evidence in pioneering studies.

Ref.	Highlights
[29]	Telehealth evaluated as an encouraging method for daily monitoring and rehabilitation in SCI, reporting successful experiences in models applied to combat-wounded individuals
[30]	NISs considered to have promising potential in support interfaces, neurological and sensory rehabilitation, diagnosis, management, and in the construction of medical knowledge around brain function
[31]	Identified significant opportunities for scientific and economic growth in the field of bioengineering for SCI support, with advancements in electronics expected to play a significant role
[32]	Predicted that the technological progress would lead to greater accessibility and affordability of ATs
[33]	Predicted that verticalization devices could offer significant benefits in reducing decubitus plagues, preventing demineralization, minimizing cardiovascular issues, and maintaining muscle tone

**Table 3:** Evidence from studies considering both the areas of intervention and the determinants of health.

Reference	Highlights
[13]	The consensus at the SCI 2020 conference assigned to the ATs 6 areas, regarding technological facilitation, prosthetic and robotic interventions and therapies across the spectrum of mild/moderate/severe”
[34]	Detected the socio-structural factors and environmental factors impacting people with SCI (including built environment, housing, transportation, assistive technology, and natural environment)

**Table 4:** Evidence from studies exploring economic impacts, social acceptance, and quality of life.

Reference	Highlights
[16]	The implementation and seating of the AT affects the ability of people with SCIs to participate equally in society. There is the need for international policies to assure equal access to resources and investigations covering multiple domains.
[19]	ATs interfacing with PCs can improve independence, participation, and self-esteem.
[22]	Investing in specific rehabilitation protocols and ATs that enhance mobility could improve health conditions and minimize costs for people with SCI.
[36]	The main needs, expectations, and barriers of people with quadriplegia and caregivers in relation to the self-help devices that are currently used for daily tasks was detected.
[37]	ATs can improve functions of the upper limbs in SCI patients, but it is challenging to draw generalizable conclusions because of a lack of investigations covering multiple domains.

**Table 5:** Evidence from studies investigating ethics and regulatory compliance.

Reference	Highlights
[12]	Discussed the potential regulatory frameworks applicable to ATs, emphasizing the intricate regulatory processes involved with particular reference to the ATs using the tongue barbell piercing.
[21]	Identified several ethical, social, and legal issues in BCIs concerning personhood, stigma, autonomy, privacy, research ethics, safety, responsibility, and justice. Few international recommendations have been developed to address these challenges.

**Table 6:** Evidence from studies regarding technological issues.

Reference	Highlights
[12]	Reported the state of the art of ATs based on the tongue barbell piercing, emphasizing their high acceptance in mechatronics integration, particularly in quadriplegia
[15]	Analyzed mechatronics and robotics as ATs for food administration, highlighting their limited use also in the most developed countries.
[17]	Advocated for the integration of virtual reality and robotics for rehabilitation to increase the efficacy of rehabilitation protocols
[18]	Suggested the use of ATs based on FES for upper extremity recovery after surgeries in tetraplegia
[20]	PEXOs for clinical applications were discussed, highlighting their potential to minimize secondary medical complications in SCI patients
[23]	Skepticism was formulated regarding the performance of PEXOs for mobility in SCI patients
[24]	Listed ATs for pressure ulcer prevention in SCI, identifying specific categories supporting self-management, but pointing out low-to-moderate effectiveness
[25]	Highlighted BCIs’ promise; however, further specific studies were suggested to gauge their applicability in the clinical setting
[26]	Reviewed electronic portable assistive devices in SCI and suggested the use of portable electronic reminders as a practice guideline
[27]	Focused on youth and identified key ATs, including therapeutic and functional stimulation, EMG biofeedback, and access of ATs to the computer
[28]	Indicated that FES therapies were useful and valuable as ATs during the subacute phase of recovery for persons with disability caused by quadriplegia
[35]	Indicated that virtual reality in home-based systems could provide efficacious therapy and facilitate the development and integration of other methods in rehabilitation
[36]	ATs were categized into: neuroprosthesis, orthotic devices, hybrid systems, robots, and arm supports
[38]	Reported that whereas changes in brain structure have been extensively studied, knowledge regarding brain connectivity following SCI is lacking; this could impact to the proper choice of an AT

## Data Availability

Not applicable.
